# Eco-Friendly Disposable WS_2_ Paper Sensor for Sub-ppm NO_2_ Detection at Room Temperature

**DOI:** 10.3390/nano12071213

**Published:** 2022-04-05

**Authors:** Daniel Matatagui, Carlos Cruz, Felix Carrascoso, Abdullah M. Al-Enizi, Ayman Nafady, Andres Castellanos-Gomez, María del Carmen Horrillo

**Affiliations:** 1 Grupo de Tecnología de Sensores Avanzados (SENSAVAN), Instituto de Tecnologías Físicas y de la Información (ITEFI), CSIC, 28006 Madrid, Spain; carlos.cruz@csic.es (C.C.); carmen.horrillo.guemes@csic.es (M.d.C.H.); 2 Materials Science Factory, Instituto de Ciencia de Materiales de Madrid (ICMM-CSIC), 28049 Madrid, Spain; felix.c@csic.es; 3 Department of Chemistry, College of Science, King Saud University, Riyadh 11451, Saudi Arabia; amenizi@ksu.edu.sa (A.M.A.-E.); anafady@ksu.edu.sa (A.N.)

**Keywords:** WS_2_, humidity sensor, nitrogen dioxide, paper-based device, room temperature sensor, 2D materials

## Abstract

We developed inexpensive and disposable gas sensors with a low environmental footprint. This approach is based on a biodegradable substrate, paper, and features safe and nontoxic electronic materials. We show that abrasion-induced deposited WS_2_ nanoplatelets on paper can be employed as a successful sensing layer to develop high-sensitivity and selective sensors, which operate even at room temperature. Its performance is investigated, at room temperature, against NO_2_ exposure, finding that the electrical resistance of the device drops dramatically upon NO_2_ adsorption, decreasing by ~42% (~31% half a year later) for 0.8 ppm concentration, and establishing a detection limit around~2 ppb (~3 ppb half a year later). The sensor is highly selective towards NO_2_ gas with respect to the interferents NH_3_ and CO, whose responses were only 1.8% (obtained for 30 ppm) and 1.5% (obtained for 8 ppm), respectively. Interestingly, an improved response of the developed sensor under humid conditions was observed (tested for 25% relative humidity at 23 °C). The high-performance, in conjunction with its small dimensions, low cost, operation at room temperature, and the possibility of using it as a portable system, makes this sensor a promising candidate for continuous monitoring of NO_2_ on-site.

## 1. Introduction

Gas sensing is becoming more and more important in our society. In fact, detection of various gases in low concentrations is crucial (and sometimes even mandatory) in fields such as air quality assessment, greenhouse gas emissions control, the quantification of volatiles for smart maintenance in the industry sector, and identification of biomarkers in medical diagnosis [1,2,3,4,5].

Among the gaseous species that should be monitored, detection of nitrogen dioxide (NO_2_) is required in different applications. In the atmosphere, NO_2_ plays the role of greenhouse gas and causes acid rain and photochemical smog problems [6]. Long-term exposure to high levels of NO_2_ produces harmful effects for humans and other living beings [7,8]. Additionally, nitrogen oxides (NO_x_) in exhaled breath are biomarkers for inflammatory and oxidative changes in lungs, serving as early indicators of the pathophysiology of many respiratory diseases [9].

Chemical sensors based on semiconductor materials [10,11,12,13,14,15], and particularly metal oxides, are the most popular devices to sense NO_2_ gas [16,17,18,19]. However, these metal-oxide-based devices present poor sensitivity at room temperature, requiring high-temperature operation that leads to high power consumption and the eventual degradation of the sensing material [20]. Moreover, metal-oxide devices require the use of substrates compatible with micro-fabrication techniques (i.e., silicon, glass, quartz, etc.), which hampers their application in disposable electronics applications where the use of ultra-low cost and biodegradable substrates is crucial to emerging technologies and environmental impacts.

Over the past two decades, thanks to the revival of interest in van der Waals materials aroused by the isolation of graphene [21], sensors based on layered materials have been presented as a real step forward in gas sensing. Their exceptionally large surface-area-to-volume ratio makes these materials strongly sensitive to adsorbed gases, and therefore they are promising candidates for gas detection [22,23,24,25,26,27,28]. In fact, over the last years, several examples of NO_2_ gas sensors based on van der Waals materials, operating even at room temperature, have been proved [26,29,30,31,32].

The attractive properties of the conventional printer paper as a substrate, mainly its environmental-friendliness and low-cost, have led researchers to develop paper-based devices for various applications, including memory devices [33], solar cells [34,35], RFID-enabled wireless sensors [36], or supercapacitors [37]. Recently, some of the authors have demonstrated the integration of van der Waals materials on paper substrates through direct abrasion against the rough surface of paper [38,39,40,41]. However, only light and temperature sensors have been demonstrated so far, with gas sensing remaining unexplored. Because of the combination of ultra-low cost, availability, and biodegradability of paper substrates, integrating van der Waals materials on paper substrates opens the door for low-cost and disposable [42,43,44,45,46,47,48,49,50] gas sensors.

Here, we demonstrate the fabrication of gas sensors on standard copy paper substrates using abrasion-induced deposited WS_2_ films as a sensing material. This process is simple to implement and yields low-cost and environmentally-friendly devices. In fact, standard copy paper substrates are biodegradable, and the sensing film (WS_2_) and electrodes (graphite) are safe, nontoxic materials that can be found as natural minerals on Earth’s crust. The sensing performance of the WS_2_-based sensor under exposure to NO_2_ gas, operating at room temperature, is examined. Furthermore, the selectivity relative to potential interfering gases (NH_3_ and CO) is analyzed [51,52,53].

## 2. Materials and Methods

### 2.1. Materials

Standard (untreated) copy printer paper (80 g/m^2^) was used as supporting substrates because of its low cost and availability. Tungsten disulfide (WS_2_) from HAGEN automation Ltd. (Bedford, UK) (0.6 microns APS Ultra Grade Micronized) was used as gas sensing channel material. Among the different semiconducting transition metal dichalcogenides, we selected WS_2_ as it yielded films with the lower electrical resistance facilitating the electrical read-out of the fabricated devices. Graphite pencil (Madrid, Spain) (4B, Faber Castell) was employed to pattern graphite-based electrical leads (it has ~80% of graphite content [54]) to connect the WS_2_ channel to the readout electronics.

### 2.2. Sensor Fabrication

**Figure 1:**
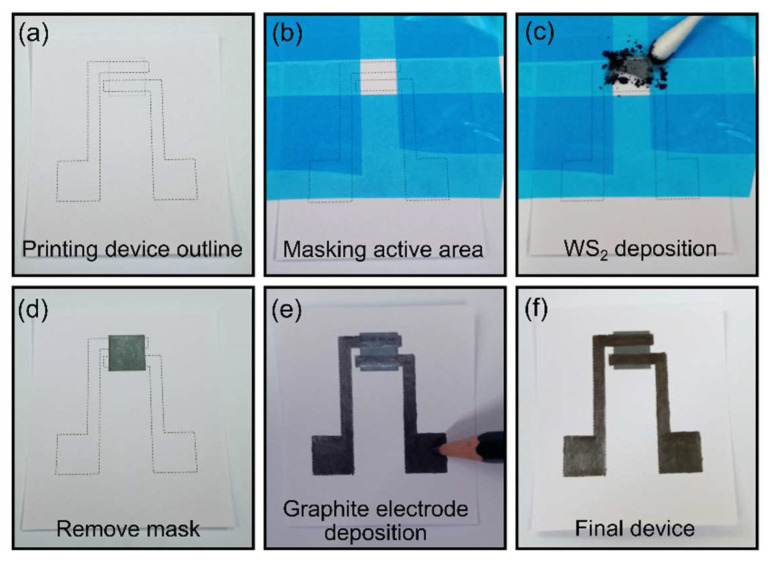
Process sequence for the sensor fabrication. (**a**) The outline of the device is printed with a standard office printer using standard copy paper. (**b**) Nitto SPV 224 tape is used to mask around the active area of the device. (**c**) An active film of WS_2_ nanoplatelets is deposited by mechanical abrasion of micronized WS_2_ powder against the paper surface. (**d**) The mask is removed, showing the patterned WS_2_ film on paper active area of the device. (**e**) Graphite electrodes are patterned by simply filling in, with a 4B graphite pencil, the area between the device outline dotted lines. (**f**) Picture of the final fabricated device.

The steps for the gas sensor fabrication are depicted in Figure 1. First, the outline of the sensitive layer channel and electrodes were printed on the paper substrate (Figure 1a). Then, a stencil mask (made of Nitto SPV 224 tape) delimited the sensitive area (Figure 1b). Micronized WS_2_ powder was rubbed against the unmasked paper substrate with a cotton swab (Figure 1c). The depositing process mimics the action of drawing/writing with a pencil on paper, where the friction forces between the van der Waals materials and paper cleaves the van der Waals crystals, leading to a network of interconnected platelets. The powder was abraded until a continuous film was reached. Then, the excess powder and the stencil mask were removed (Figure 1d). In the last process step, graphite electrodes were deposited on top of the sensitive material by drawing directly with a high-graphite content pencil (Figure 1e). These electrodes were contacted with spring-loaded probes (pogo pins) integrated inside the test chamber. Figure 1f shows a picture of the final device.

### 2.3. Material Characterization

Scanning electron microscopy (SEM) and energy dispersive X-ray spectroscopy (EDX), using a FE-SEM, FEI Nova NANOSEM 230 (Hillsboro, OR, USA), were used to characterize the morphology and the composition of the WS_2_ films deposited on paper. An electron energy of 7 keV was employed for imaging and 14 keV for EDX spectroscopy.

### 2.4. Experimental Setup

**Figure 2:**
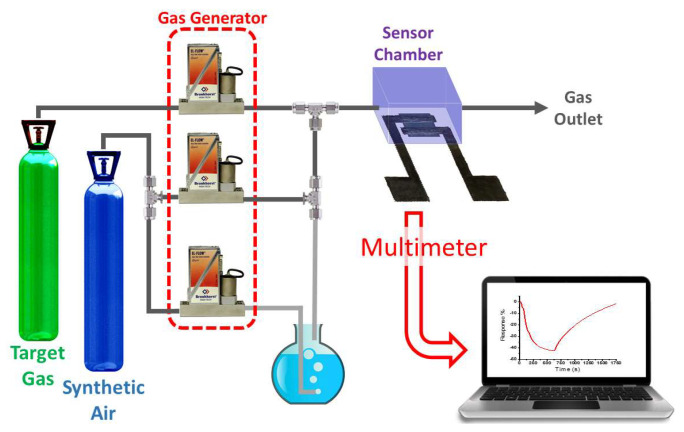
Experimental setup used to measure different gases concentrations with the WS_2_-on-paper sensor in real time.

The chemoresistive sensor was placed inside a 6.25 mL volume airtight chamber for its characterization in different reducing and oxidizing atmospheres. Airflow inside the chamber was set to 100 mL/min, switching between gas sample for 10 min (exposition time) and synthetic air for 20 min (purge time). Gas cylinders supplied target gases with appropriate concentrations and balanced with the carrier gas (synthetic air): NO_2_ (1 ppm), CO (10 ppm), and NH_3_ (50 ppm) (all of them from Nippon Gases). Then, the initial sample concentration was diluted with synthetic air by using a gas mixing unit (GMU, Ray IE, Cáceres, Spain)to obtain the required exposed concentration. For proper control of the relative humidity (RH) inside the chamber, a handheld thermohygrometer RS1364 was used. The temperature was kept at 23 °C during the tests, and the required RH was achieved with a third flow controller that regulates the synthetic air bubbling through deionized water (Figure 2).

The sensor was kept at room temperature while variations of the resistance over time were recorded with a digital multimeter (Keithley 2001). The experiment control and real time data acquisition was implemented with a PC using an in-house custom-made software developed with LabVIEW. The response of the sensor was calculated with the following equation:

(1) $$Response=\Delta R/R_{0}=\left(R-R_{0}\right)/R_{0}$$

where R is the electrical resistance for the sensor in the tested gas and $R_{0}$ is the resistance of the sensor in the air.

## 3. Results

### 3.1. Structural and Morphological Characterization

**Figure 3:**
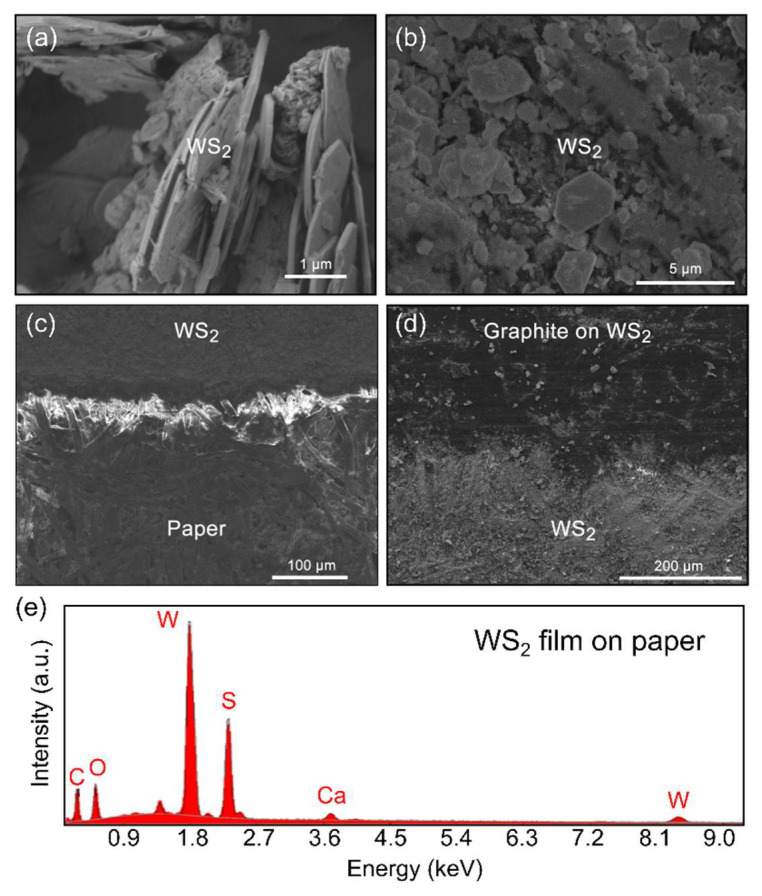
SEM images of (**a**) the cross-section of the WS_2_ flakes deposited on paper by abrasion technique, (**b**) the network of WS_2_ flakes deposited on paper after abrasion-induced deposition, (**c**) the interface between bare paper and the deposited WS_2_ film, and (**d**) the border between the surface of the WS_2_ sensing area and the WS_2_ covered by the graphite electrode. (**e**) EDX spectrum for the micronized WS_2_ deposited on the paper.

Figure 3a shows SEM image of the cross-section of the WS_2_ flakes forming the micronized WS_2_ powder. The flakes are initially 5–10 µm in lateral size and 50–150 nm thick. Figure 3b shows a SEM image of the porous microscopic structure of the WS_2_ film deposited onto the paper substrate, formed by interconnected crystalline WS_2_ platelets, ensuring a very large effective surface area of the device. During the abrasion process, the WS_2_ flakes are cleaved, reducing their lateral dimensions to 1–5 µm and their thickness to sub-50 nm. Figure 3c shows a low magnification SEM image of a WS_2_ film obtained after its deposition on the paper substrate. The bare paper has fibrous-like structures arising from the cellulose fibers. The abrasion-induced deposition method yielded a continuous film of packed WS_2_ platelets covering the fibers. The bare paper and WS_2_ film can be easily distinguished because of their different contrast under SEM inspection. Figure 3d shows a SEM image of the sensitive area/electrode interface where it can be observed a sizable change in contrast due to the difference in electrical conductivity between the WS_2_ film and the WS_2_ film covered with graphite. The chemical composition of the film was characterized by energy dispersive X-ray (EDX) spectroscopy. Apart from the prominent W and S peaks, expected from the WS_2_ film, the spectrum had peaks associated with the presence of C and O, arising from the paper substrate. The spectrum also showed a Ca peak, attributed to the presence of calcium carbonate, a filler usually added to paper pulp to achieve a brighter white color (Figure 3e).

### 3.2. Electrical Characterization

A thorough characterization of the electrical properties of abrasion-induced deposited WS_2_ films on copy paper can be found in Ref [41]. Briefly, the resistivity of the films, determined through current vs. voltage measurements in transfer length configuration, ranges from ~360 Ω·m to ~530 Ω·m and electric field effect measurements demonstrated the p-type character of the WS_2_ film. Additionally, in the mentioned reference, 118 devices were developed to study the reproducibility, showing a low dispersion taking into account the nature of the films: a random network of interconnected platelets where percolation transport is expected.

The sensor was kept at air atmosphere, and after a few minutes, the calculated root-mean square (RMS) noise level was approximately 0.01% for the sensor. Thanks to the electrical continuity of the WS_2_ film, the device operates with low noise that is a consequence of the dry deposition method, and a good adhesion between sensitive material and paper fibers.

### 3.3. Gas Sensor Characterization

**Figure 4:**
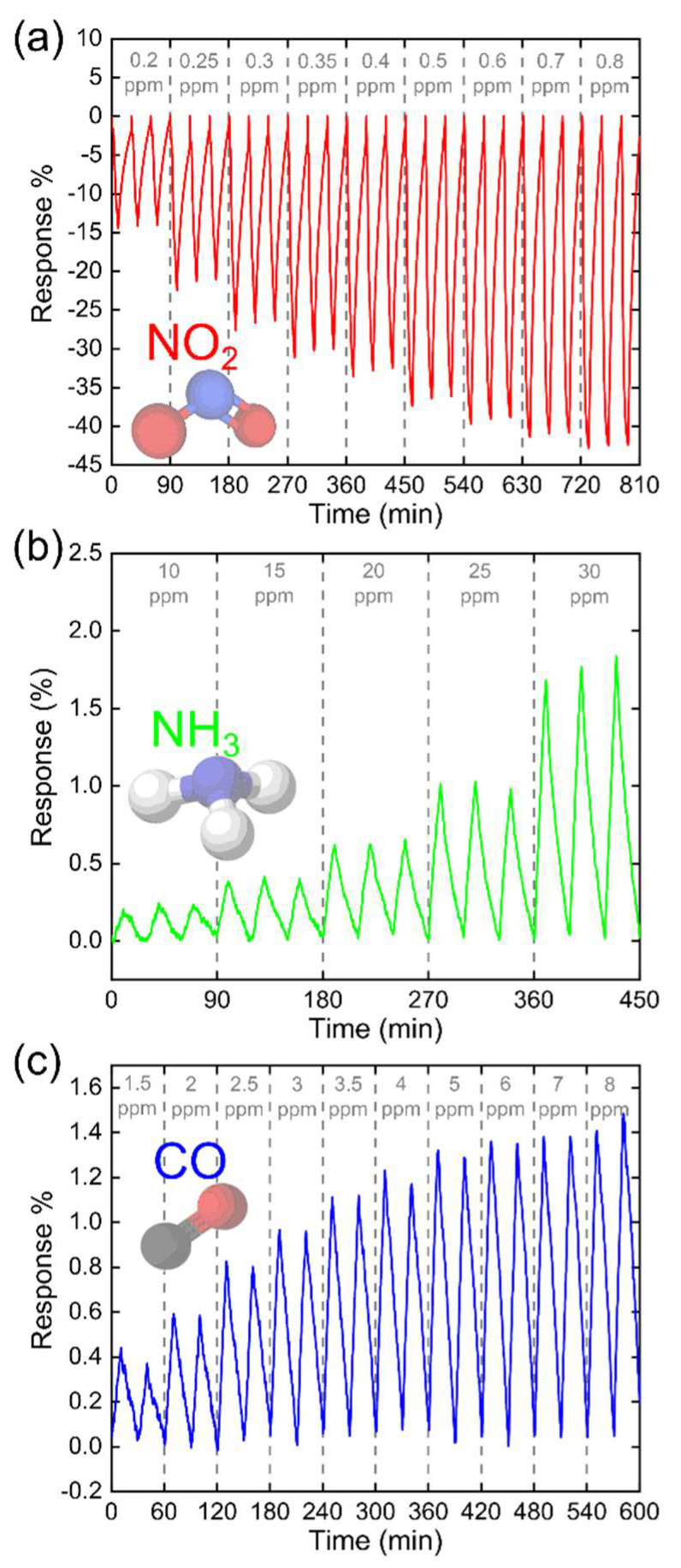
Paper-based sensor response to cyclic exposure to (**a**) NO_2_, (**b**) NH_3_, and (**c**) CO gases.

To characterize the performance of this sensor, its sensitivity, and response time, we studied the changes in resistance upon cyclic exposition and purge processes with NO_2_ at various concentrations ranges (0.2 ppm–0.8 ppm, see Figure 4a). The gas sensing mechanism is attributed to the surface reactions between the p-type WS_2_ platelets and gas molecules. In the case of a p-type semiconductor in an oxidant environment (NO_2_), the concentration of electrons on the surface decreases (the number of holes increases) and, consequently, the resistance of the WS_2_ film decreases (Figure 4a). The sensor device showed a fast recovery with a low baseline drift of 0.6% at 0.8 ppm of NO_2_. Therefore, an automatic baseline subtraction method based on linear correction for measurements before exposition and in the final of the purge time was implemented.

**Figure 5:**
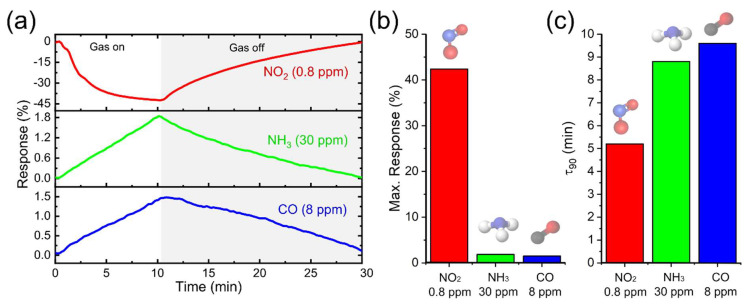
(**a**) Real time responses, (**b**) maximum (absolute value) responses and (**c**) response time, τ_90_, of the paper-based sensor to 0.8 ppm of NO_2_ (red), 30 ppm of NH_3_ (green), and 8 ppm of CO (blue).

In most real-life applications, the target gas is in a complex environment surrounded by several gases at different concentrations, requiring sensors with high sensitivity and selectivity to discriminate and classify the target gas. Important interfering gases, in the above applications, are carbon monoxide (CO) and ammonia (NH_3_) [55,56]. Therefore, gas sensors with negligible interference between reducing and oxidizing environments, i.e., a high absolute selectivity, are highly desirable to achieve a more reliable signal interpretation. To test the selectivity of the WS_2_ on paper NO_2_ sensor, we have subjected the device to cyclic exposition and purge processes with CO and NH_3_ at various concentrations ranges (1.5 ppm–8 ppm for CO and 10 ppm–30 ppm for NH_3_, see Figure 4b,c). Upon exposure to CO and NH_3_, the resistance increases as expected for a p-type semiconductor, because the generated electrons recombine with holes. The gas test showed the sensor has a remarkably higher sensitivity towards NO_2_ (42% resistance change at 0.8 ppm) than NH_3_ and CO, whose responses were 1.8% (obtained at 30 ppm) and 1.5% (obtained at 8 ppm), respectively (see Figure 5a,b). This can be justified by the adsorption kinetics of gas molecules on the sensitive material. Additionally, the high sensitivity and selectivity to NO_2_ is consistent with results of density functional theory calculation in Ref. [32]. Interestingly, this article explains that the chemically reactive edge sites of WS_2_ served as highly favorable active sites for direct interaction with target NO_2_ gas molecules. This is consistent with the fact that abrasion-induced is an effective method to generate numerous edge sites in deposited WS_2_ nanoplatelets on paper, since the technique induces fracturing, tearing, and peeling off from substrates.

Accordingly, the response speed of the device was studied. We determined the response time parameter τ_90_, defined as the time necessary to reach approximately 90% of the response when the sensor is subjected to an abrupt change in atmosphere. The τ_90_ values obtained were NO_2_ 5.2 min at 0.8 ppm, NH_3_ 8.8 min at 30 ppm, and CO 9.6 min at 8 ppm (Figure 5c). In comparison, the paper-based sensor showed the shortest response time for NO_2_ that nearly achieved the equilibrium. In contrast, CO and NH_3_ responses had not yet approached an equilibrium, resulting in a high sensitivity NO_2_ gas sensor with insignificant NH_3_/CO-interference. Therefore, this very high selectivity with respect to potential interfering gases of the sensor is highly advantageous to be used for gas sensing applications.

The structural continuity of the micronized WS_2_ particles deposited by abrasion provides higher electrical conductivity toward a lower limit of detection (LOD) compared to sensors fabricated by other methods, such as drop-casting [26]. From the response for 0.8 ppm of NO_2_, a theoretically achievable LOD of around 2 ppb was calculated, which is equivalent to a signal-to-noise ratio (SNR) value of three.

**Figure 6:**
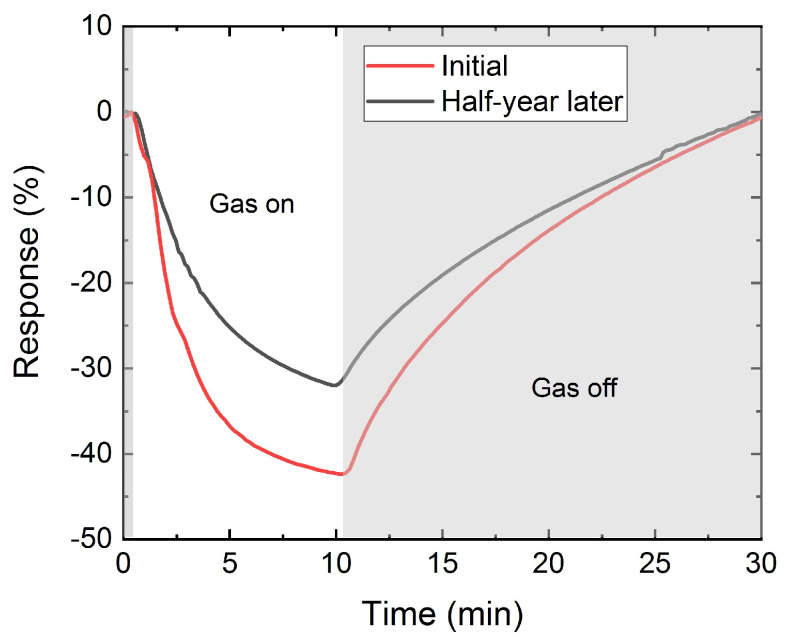
Real time response of the sensor obtained with NO_2_ sample at the concentration of 0.8 ppm for the as-fabricated device (red) and after half a year of exposure to ambient conditions (black).

**Figure 7:**
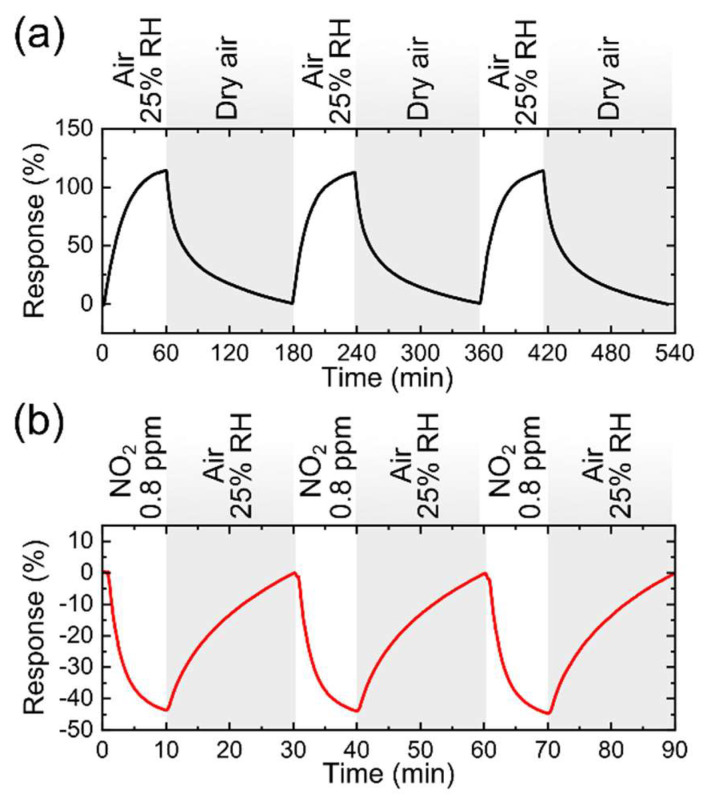
Real time response of the paper-based sensor tested at 23 °C (**a**) with intervals of dry air (0% RH) and 25% RH (**b**) for 0.8 ppm of NO_2_ in an environment with RH 25% at 23 °C.

In order to assess the stability of these devices upon environmental degradation, we performed a new set of measurements half a year after its fabrication (the sensor was stored under ambient conditions during that time). Thereafter, the response for 0.8 ppm of NO_2_ was slightly decreased to 31%, increasing the LOD around 3 ppb (Figure 6). In particular, the decrease of the gas response is small between measurements for a half year interval, which demonstrates that the paper-based sensor has a slow degradation, maintaining a high response over time. The effect of relative humidity on the paper-based sensor was tested at 23 °C with 25% RH and it responded efficiently to humidity, obtaining a maximum response of 114% (Figure 7a). Cross-sensitivity measurements were carried out to assess the influence of RH on the sensor response to NO_2_. Figure 7b illustrates the effect of 0.8 ppm of NO_2_ detection in an environment with a RH of 25% with a sensor response of ~44%.

**Figure 8:**
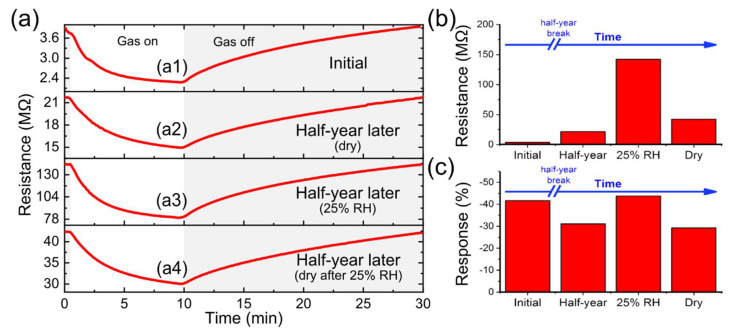
(**a**) Real time response of the sensor obtained with NO_2_ sample at the concentration of 0.8 ppm (**a1**) in a dry environment a few days later than the sensor fabrication, (**a2**) in dry environment half a year later than the sensor fabrication, (**a3**) in an environment with RH 25% at 23 °C, and (**a4**) in the dry environment after humidity exposition. Values of (**b**) level of the sensor resistance and (**c**) sensor response under the different operating conditions.

The experimental responses of NO_2_ over time and under humid conditions were compared for 0.8 ppm (Figure 8a1–a4). In the days following device manufacture, the sensor had a high response close to 42% with an operating resistance of ~4 MΩ. Then, after half a year where the sensor was stored in ambient conditions, the resistance increased to ~22 MΩ, decreasing the response to ~31%, which was attributed to the effects of sensor poisoning by gases surrounding in ambient during the half a year period. However, a positive effect of humid conditions (25% RH at 23 °C) is that at the same gas concentration the sensor response increased to 44%, simultaneously the resistance scaled up ~142 MΩ. The improved response to NO_2_ with humidity can be justified by the intrinsic and induced dipole moments of the molecules and their intermolecular charge transfer [57]. Finally, there was a practically total regeneration of the sensor after humidity exposition was obtained for dry synthetic air, and the sensor showed a response of ~30% for a resistance operation of ~42 MΩ (Figure 8b,c). Therefore, the sensor performance is a huge benefit since it can work on a large range of tests for multidisciplinary applications carried out in humid conditions with sensitivity gain.

## 4. Conclusions

In summary, we fabricated and characterized a disposable NO_2_ sensor based on a *p*-type WS_2_ film on standard paper. The sensing film was deposited by a low-cost and easy to implement abrasion-induced method, establishing a nanostructured sensitive layer by exfoliation of micronized WS_2_ particles and an electrical connection among flakes. The structure of the WS_2_ sensing film was characterized by using SEM, which showed rough and porous film formed by interconnected WS_2_ flakes. The sensor showed excellent sensing properties at room temperature with a response higher than 42% (31% half a year later) at 0.8 ppm NO_2_ and with a significant LOD of around 2 ppb (3 ppb half a year later). The relative humidity of 25% at 23 °C has a beneficial impact. The result indicates the high sensitivity, selectivity, and repeatability of the presented sensor towards sub-ppm level of NO_2_ gas, which makes it a promising candidate for monitoring of NO_2_ sensing.

## Data Availability

The data presented in this study are available on request from the corresponding authors.
